# 1787. County Level Association Between the Rate of Fluoroquinolone-Resistance Gram Negative Bacteria and Fluoroquinolone Utilization in Kansas

**DOI:** 10.1093/ofid/ofac492.1417

**Published:** 2022-12-15

**Authors:** Robert D Fulmer, Kellie Wark

**Affiliations:** Spectrum Health, Grand Rapids, Michigan; University of Kansas, Kansas City, Kansas

## Abstract

**Background:**

Kansas ranks as the 8th-worst state nationally for outpatient antibiotic prescribing, with a rate of 690 antibiotic prescriptions per 1000 population. The statewide rate of fluoroquinolone (FQ) is disproportionately higher than other antibiotic drug classes. Statewide antibiograms can be developed by health departments for antimicrobial stewardship monitoring. The purpose of this study was to investigate the correlation between the FQ resistance rate of gram-negative bacteria to FQ consumption.

**Methods:**

A retrospective design utilizing 2018-2019 county level FQ-resistant *Escherichia coli, Klebsiella pneumoniae* and *Pseudomonas aeruginosa* was categorized by county level based on the Kansas statewide antibiogram. Outpatient FQ utilization data was obtained from IQVIA and categorized into county level FQ rate based on prescriptions dispensed per 1000 population. Linear regression was used to compare antibiotic resistance (AR) to FQ utilization by county.

**Results:**

Sixty-six of 123 facilities contacted for antibiogram data responded with results. Of these, 47 were critical access hospitals, and the rest were acute care hospitals or clinics. A total of 31,573 *E. coli* isolates, 5,942 *K. pneumoniae* and 5,017 *P. aeruginosa* isolates were represented. All 106 counties had FQ prescription data. Of the 49 counties with AR reported, FQ rates ranged from 4.6 to 105.6/1000 population. There was no statistical significance associated between counties for FQ-resistant *E. coli, K. pneumoniae* or *P. aeruginosa* with FQ-utilization rates (p=0.184, 0.572, 0.352, respectively). Comparing urban and rural regions was also compared for each AR-FQ use combination. There was statistical significance for urban *P. aeruginosa* FQ-resistance and FQ utilization (p=0.025), but not for urban *E. coli* (p=0.120) or urban *K. pneumoniae* (p=0.331).

**Conclusion:**

A strong correlation was demonstrated between FQ consumption and rates of FQ-resistant *P. aeruginosa* within urban counties. No significant correlations were found between FQ consumption rates and other organisms. This study demonstrates additional applications for state-level antibiogram data, particularly when paired with antibiotic use data.

**Disclosures:**

**All Authors**: No reported disclosures.

